# Intracapsular injection of triple-drug solution in the treatment of early and mid-stage knee osteoarthritis

**DOI:** 10.3389/fsurg.2026.1756840

**Published:** 2026-02-24

**Authors:** Fulin Li, Tingyou Ning, Yingrong Mo, Xiao Huang, Wenhui Liu, Dong Yin

**Affiliations:** 1 Jinan University, Guangzhou, China; 2 Department of Joint Surgery and Sports Medicine, the People’s Hospital of Guangxi Zhuang Autonomous Region, Guangxi Academy of Medical Sciences, Nanning, China; 3 Department of Orthopedics, The First People’s Hospital of Qinzhou, Qinzhou, Guangxi, China; 4 Department of Pharmacy, the People’s Hospital of Guangxi Zhuang Autonomous Region, Guangxi Academy of Medical Sciences, Nanning, China

**Keywords:** betamethasone, function, hyaluronic acid sodium, KOA, triple injection

## Abstract

**Objective:**

The objective of this study was to evaluate the efficacy and safety of intra-articular injection of the “triple injection” in the treatment of early and middle-stage knee osteoarthritis (KOA).

**Materials and methods:**

A total of 120 patients with unilateral KOA, recruited from October 2021 to December 2023, were randomly divided into two groups with 60 cases in each group. The control group received intra-articular injection of 2 mL sodium hyaluronate once a week for 5 consecutive weeks. The experimental group received intra-articular “triple injection” (0.3 mL betamethasone + 0.7 mL lidocaine + 2 mL sodium hyaluronate) in the first week, followed by intra-articular injection of 2 mL sodium hyaluronate once a week for 4 consecutive weeks. The clinical efficacy was evaluated using the Western Ontario and McMaster Universities Osteoarthritis Index (WOMAC), Visual Analogue Scale (VAS), Hospital for Special Surgery (HSS) knee score, and flexion range of motion (ROM) before treatment, as well as 1 week, 4 weeks, 12 weeks, and 24 weeks after treatment.

**Results:**

Comparisons of WOMAC scores, VAS scores, HSS scores, and ROM before treatment revealed no statistically significant differences between the two groups (all *P* > 0.05). In contrast, statistically significant differences in WOMAC scores, VAS scores, HSS scores, and ROM between the two groups were observed at different time points after treatment (all *P* < 0.05). Additionally, the comparison of overall efficacy in K-L grade III patients between the two groups showed a statistically significant difference (*P* < 0.05), and no complications were observed in any of the patients.

**Conclusion:**

Intra-articular injection of sodium hyaluronate and the “triple injection” are both effective therapeutic modalities for the early and mid-stage of KOA. Compared with sodium hyaluronate, the “triple injection” can more effectively relieve pain and improve knee joint function.

**Clinical trial registration:**

Identifier ChiCTR2100048131 with a registration date of 04/07/2021.

## 1 Introduction

Knee osteoarthritis (KOA) is a common bone and joint disorder characterized by gradual cartilage degeneration, often accompanied by local pain, joint deformity, swelling, and motor impairment (1). Due to the complexity of KOA, there is still no effective treatment to date (2). For KOA, the primary treatment goal is to relieve pain, improve joint function, slow disease progression, and enhance patients' quality of life (3). The management of KOA encompasses basic treatment, pharmacotherapy, and surgical intervention, with intra-articular drug injection typically reserved for cases where oral or topical medications yield insufficient therapeutic effects (4). Compared with systemic administration, intra-articular delivery offers numerous advantages, including improving local bioavailability, reducing systemic exposure and the incidence of adverse reactions, and lowering treatment costs (5).

In recent years, intra-articular injection of multi-drug regimens for KOA treatment has garnered considerable attention. The involved drugs or pharmaceutical preparations encompass sodium hyaluronate, glucocorticoids, platelet-rich plasma, ozone, local anesthetics, and vitamins (6). Additionally, tension in the nerves and muscles surrounding the knee joint is another key contributor to pain (6). Notably, there are no unified standards for drug formulation and dosage, and the clinical efficacy remains to be further validated. This study aims to assess the clinical efficacy and safety of the “triple injection” (0.3 mL betamethasone +0.7 mL lidocaine +2 mL sodium hyaluronate) regimen.

## 2 Methods

### 2.1 Patients and design

This study was conducted at the People's Hospital of Guangxi Zhuang Autonomous Region (Guangxi Academy of Medical Sciences) from October 2021 to December 2023 and approved by the hospital's Ethics Committee. The study was designed as a single-blind, prospective randomised controlled trial (RCT; Chinese Clinical Trial Registry No.: ChiCTR2100048131; registration date: 07 April 2021).

Inclusion criteria: (1) Aged 50–80 years; (2) Meets the diagnostic criteria for KOA established by the American College of Rheumatology (ACR) (7); (3) Kellgren & Lawrence (K-L) classification grade II or III for KOA (8); (4) Provides signed informed consent.

Exclusion criteria: (1) I Abnormal rheumatologic or immunologic indices; (2) Ligament injuries or a history of knee trauma; (3) Joint mouse or meniscal tears resulting in joint locking; (4) Patellofemoral arthritis; (5) Use of analgesics within the past 1 week, or intra-articular injections or biological agents administered within the past 6 months; (6) Previous knee surgery or physical therapy within the past 6 months; (7) Severe cardiovascular/cerebrovascular diseases, tumors, diabetes mellitus, active infections, or immunosuppressive conditions; (8) Anticipated treatment interruption or high risk of loss to follow-up.

**Figure 1:**
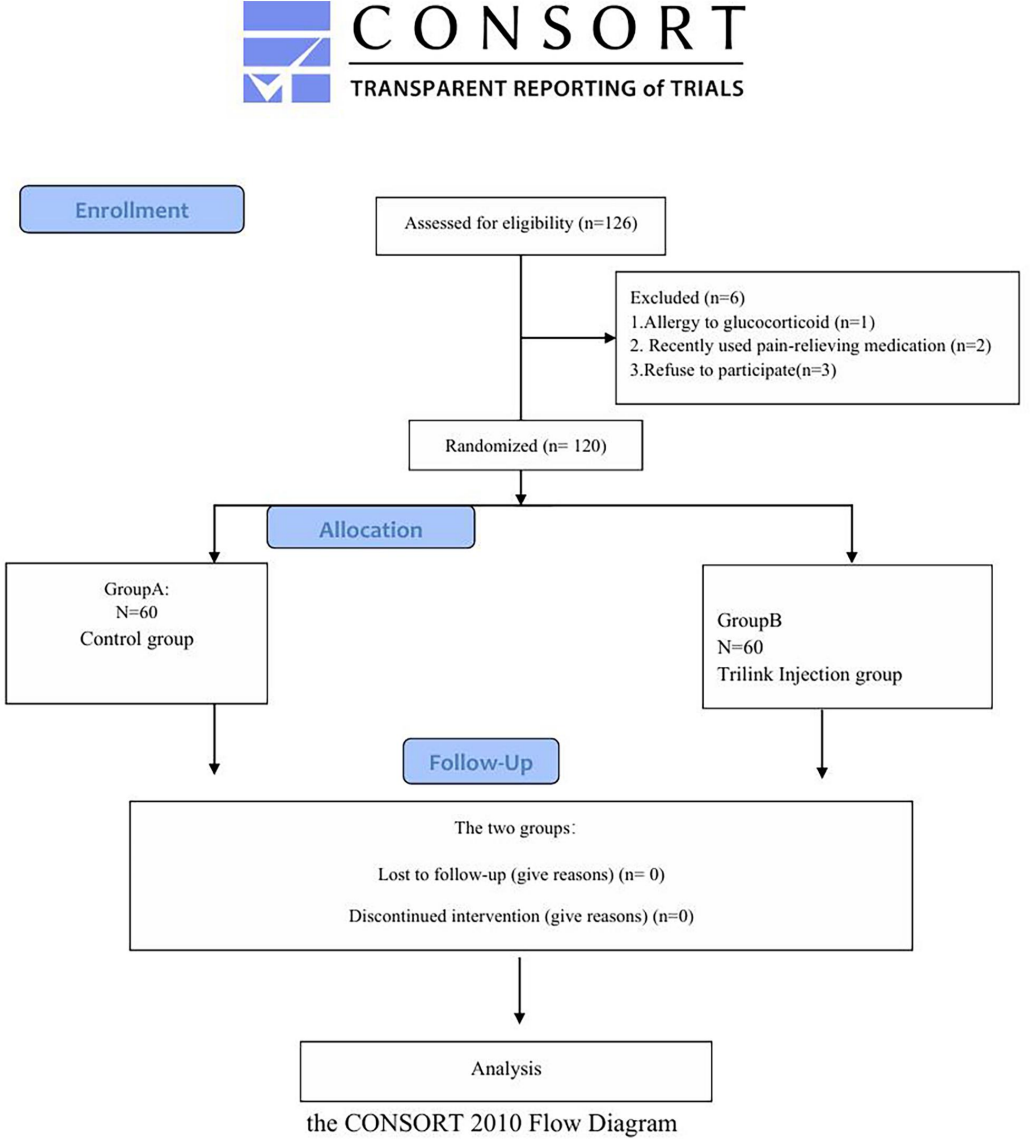
Schematic diagram of the patient study process.

This study complied with the ethical principles outlined in the World Medical Association's Declaration of Helsinki (2013) (9). All participants were sequentially numbered based on the order of their enrollment, and their randomization assignments were placed in opaque, sealed envelopes, which were opened immediately prior to the initiation of the intervention. Patients were unaware of the specific medication regimen they received (Figure 1). All participants provided written informed consent prior to study initiation. The study was approved by the Ethics Committee of the People's Hospital of Guangxi Zhuang Autonomous Region.

### 2.2 Operation management

Patients in the control group (Group A) were placed in a supine position with the affected knee fully extended to fully expose the injection site. The injection point was selected as the intersection of the superior and lateral borders of the patella. Routine disinfection was performed with iodine tincture followed by alcohol, and sterile draping was applied. The operator wore sterile gloves and performed puncture using a 5 mL sterile syringe. Synovial fluid in the joint cavity was drained as much as possible, after which the operator injected 2 mL of sodium hyaluronate into the joint cavity. Following needle withdrawal, the puncture site was pressed with a sterile cotton swab, covered with sterile gauze, and the patient was assisted in performing knee flexion and extension exercises. Patients were instructed to keep the puncture site dry for 48 h post-injection and to receive regular weekly treatment for a total of 5 weeks.

The procedural steps for patients in the experimental group (Group B) were identical to those of the control group both pre- and post-intra-articular injection. Patients in Group B received a single “intra-articular triple injection” (0.3 mL betamethasone + 0.7 mL lidocaine + 2 mL sodium hyaluronate) in Week 1, followed by weekly intra-articular injections of 2 mL sodium hyaluronate at Weeks 2, 3, 4, and 5. All intra-articular injections in both groups were performed by the same physician.

### 2.3 Outcome assessment

This study aimed to evaluate the clinical efficacy and safety of the “triple injection” by comparing WOMAC, HSS, and VAS scores, knee flexion range of motion (ROM), as well as adverse reactions and complications between the two groups at 1, 4, 12, and 24 weeks post-treatment.

### 2.4 Postoperative complications

Patients were assessed for complications including injection site infections, nausea and vomiting, and cartilage injuries. Injection site infections were primarily evaluated based on injection site conditions, such as erythema, swelling, warmth, and tenderness. For cartilage injuries, the Magnetic Resonance Observation of Cartilage Repair Tissue (MOCART) (10, 11) scoring system was used. Based on seven relevant parameters, this system provides a standardized evaluation of cartilage repair morphology. The scoring system comprises seven items (total score: 100 points): cartilage/bone defect volume filling (20 points), integration with adjacent tissues (20 points), repaired tissue surface integrity (5 points), repaired tissue signal intensity (15 points), bone defect or bone spicule presence (20 points), bone marrow edema-like signal (10 points), and subchondral bone cyst formation (10 points). A higher MOCART score indicated a more favorable cartilage repair outcome.

### 2.5 Sample size calculation

The primary outcome measure was the Visual Analogue Scale (VAS) score during walking at 1 week post-treatment. It was estimated that the mean VAS score would differ by 0.95 points between the experimental and control groups [with a common standard deviation (SD) based on preliminary data]. With a statistical power of 90% (1−*β* = 0.90) and a two-sided significance level (*α*) of 0.05, 53 patients per group were required. To account for an anticipated dropout rate of 10%–15%, the sample size was adjusted upward. Consequently, 60 patients were enrolled in each group, resulting in a total sample size of 120 patients.

### 2.6 Statistical analysis

All statistical analyses were performed using SPSS 27.0 software (IBM Corp., Armonk, NY, USA). Normally distributed data were expressed as mean ± standard deviation (x¯ ± s), and intergroup differences were compared using the independent samples *t*-test. Data with a non-normal distribution were expressed as median [interquartile range (IQR), Q25%–Q75%], and intergroup comparisons were conducted using the Mann–Whitney *U* test. Repeated-measures data were presented as mean ± standard deviation (x¯ ± s), mean differences, and 95% confidence intervals (CIs), and were analyzed using the generalized estimating equation (GEE) to account for repeated measurements. Categorical data were expressed as constituent ratios or rates (%), with intergroup comparisons performed using the Chi-square (*χ*^2^) test or Fisher's exact test (for small sample sizes). Ordinal data were analyzed using the Mann–Whitney *U* test. Statistical significance was set at *α* = 0.05, with *P* < 0.05 considered statistically significant.

## 3 Results

### 3.1 Patient demographics

Baseline characteristics including gender, age, body mass index (BMI), disease duration, Kellgren-Lawrence (K-L) classification (Grades II and III), and comorbidities (hypertension, diabetes mellitus, coronary heart disease) were comparable between the two groups (all *P* > 0.05; Table 1).

**Table 1:** Comparison of patient demographics between two groups.

Demographics	Group A (*N* = 60)	Group B (*N* = 60)	t/*χ*^2^/Z	*P*
Gender (*n*, %)				
Male	8 (13.3)	14 (23.3)	1.002	0.506*
Female	52 (86.7)	46 (76.7)		
Age	61.03 ± 7.04	61.53 ± 8.025	−0.256	0.798^#^
BMI	24.71 ± 3.11	23.82 ± 1.50	1.401	0.168^#^
(kg/m^2^)				
Process(month)	28.10 ± 8.49	29.43 ± 9.66	−0.568	0.572^#^
K-L (*n*, %)				
Ⅱ	38 (63.3)	34 (56.7)	0.523	0.601^&^
Ⅲ	22 (36.7)	26 (43.3)		
Comorbidities				
Hypertension (*n*, %)			0.073	0.787*
No	40 (66.7)	38 (63.3)		
Yes	20 (33.3)	22 (36.7)		
Diabetes (*n*, %)				
No	48 (80.0)	44 (73.3)	0.373	0.542*
Yes	12 (20.0)	16 (26.7)		
CHD (*n*, %)				
No	58 (96.7)	54 (90.0)	1.071	0.301*
Yes	2(3.3)	6(10.0)		

CHD, coronary heart disease.

# *T* test.

* χ^2^ test.

^&^ Mann–Whitney *U* test.

### 3.2 Comparison of WOMAC between two groups before and after treatment

Generalized estimating equation (GEE) analysis revealed a significant interaction effect for intergroup comparisons of WOMAC scores, indicating that the trajectory of WOMAC score changes differed between the two groups over time. Pairwise comparisons of simple effects showed that prior to treatment, the mean difference [95% confidence interval (CI)] in WOMAC scores between the control and experimental groups was 0.20 (−3.02, 3.42) (*P* = 0.903), with no statistically significant intergroup difference. At 1, 4, 12, and 24 weeks post-treatment, the mean differences (95% CIs) in WOMAC scores favoring the experimental group were 6.57 (4.17, 8.97), 15.20 (12.43, 17.97), 10.47 (7.93, 13.00), and 11.03 (7.98, 14.09), respectively (all *P* < 0.05), demonstrating statistically significant intergroup differences (Table 2).

**Table 2:** GEE analysis of WOMAC scores in both groups before and after treatment.

Point-in-time	Group A (*N* = 60)	Group B (*N* = 60)	Differentials (95% CI)	χ^2^	*P*
Before treatment	55.87 ± 6.12	55.67 ± 6.81	0.20 (−3.02–3.42)	0.015	0.903
1 week	42.73 ± 4.79A	36.17 ± 4.85Aa	6.57 (4.17–8.97)	28.789	<0.001
4 weeks	28.97 ± 6.06AB	13.77 ± 5.04ABa	15.20 (12.43–17.97)	115.499	<0.001
12 weeks	23.80 ± 5.77ABC	13.33 ± 4.33ABa	10.47 (7.93–13.00)	65.318	<0.001
24 weeks	21.47 ± 7.16ABCD	10.43 ± 4.90ABCDa	11.03 (7.98–14.09)	50.146	<0.001
χ^2^	5768.65	11,054.1			
*P*	<0.001	<0.001			
Overall test					
Group Point-in-time	χ^2^ = 42.36, *P* < 0.001 χ^2^ = 12,629.33, *P* < 0.001				
group* point-in-time	χ^2^ = 382.85, *P* < 0.001				

(A) Compared with pre-treatment, *P* < 0.05; (B) Compared with 1 week after treatment, *P* < 0.05; (C) Compared with 4 weeks after treatment, *P* < 0.05; (D) Compared with 12 weeks after treatment, *P* < 0.05; (a) Compared with the control group, *P* < 0.05; All pairwise comparisons were adjusted using the Bonferroni correction.

GEE, generalized estimating equations model; χ^2^, Wald Chi-square.

### 3.3 Comparison of VAS scores between two groups before and after treatment

GEE analysis indicated a significant interaction effect for intergroup comparisons of VAS scores, reflecting that the trend of pain relief (VAS score reduction) differed between the two groups over time. Pairwise comparisons of simple effects showed that prior to treatment, the mean difference [95% confidence interval (CI)] in VAS scores between the control and experimental groups was −0.25 (−0.56, 0.07) (*P* = 0.125), with no statistically significant intergroup difference. At 1, 4, 12, and 24 weeks post-treatment, the mean differences (95% CIs) in VAS scores favoring the experimental group were 0.99 (0.52, 1.47), 1.66 (1.24, 2.09), 1.26 (0.87, 1.64), and 1.47 (1.06, 1.88), respectively (all *P* < 0.05), confirming statistically significant intergroup differences (Table 3).

**Table 3:** GEE analysis of VAS scores in both groups before and after treatment.

Point-in-time	Group A (*N* = 60)	Group B (*N* = 60)	Differentials (95% CI)	χ^2^	*P*
Before treatment	6.21 ± 0.63	6.46 ± 0.64	−0.25 (−0.56–0.07)	2.351	0.125
1 week	4.90 ± 0.80A	3.91 ± 1.09Aa	0.99 (0.52–1.47)	16.740	<0.001
4 weeks	3.39 ± 0.92AB	1.73 ± 0.78ABa	1.66 (1.24–2.09)	58.723	<0.001
12 weeks	2.78 ± 0.88ABC	1.53 ± 0.67ABCa	1.26 (0.87–1.64)	39.985	<0.001
24 weeks	3.02 ± 0.95ABC	1.55 ± 0.69ABCa	1.47 (1.06–1.88)	48.616	<0.001
χ^2^	3,843.842	83,680.536			
* P*	<0.001	<0.001			
Overall test					
Group point-in-time	χ^2^ = 33.53, *P* < 0.001 χ^2^ = 17,997.19, *P* < 0.001				
Group* point-in-time	χ^2^ = 929.60, *P* < 0.001				

(A) Compared with pre-treatment, *P* < 0.05; (B) Compared with 1 week after treatment, *P* < 0.05; (C) Compared with 4 weeks after treatment, *P* < 0.05; (D) Compared with 12 weeks after treatment, *P* < 0.05; (a) Compared with the control group, *P* < 0.05; All pairwise comparisons were adjusted using the Bonferroni correction.

GEE, generalized estimating equations model; χ^2^, Wald Chi-square.

### 3.4 Comparison of HSS scores between two groups before and after treatment

GEE results revealed an interaction effect when comparing Hospital for Special Surgery (HSS) scores. Subsequent pairwise comparisons of simple effects showed no statistically significant difference in HSS scores between the two groups before treatment: the mean difference was −0.03 [95% confidence interval (CI): −4.32–4.26, *P* = 0.988]. However, statistically significant between-group differences in HSS scores were noted at 1 week, 4 weeks, 12 weeks, and 24 weeks after treatment. Specifically, the mean differences (95% CIs) at these time points were −5.27 (−9.48 to −1.06), −5.87 (−9.68 to −2.05), −10.23 (−12.69 to −7.77), and −10.20 (−13.00 to −7.40), respectively, with all *P*-values < 0.05 (Table 4).

**Table 4:** GEE analysis of HSS scores in both groups before and after treatment.

Point-in-time	Group A (*N* = 60)	Group B (*N* = 60)	Differentials (95% CI)	χ^2^	*P*
Before treatment	61.80 ± 9.13	61.83 ± 8.09	−0.03 (−4.32–4.26)	<0.001	0.988
1 week	67.10 ± 7.35A	72.37 ± 9.43Aa	−5.27 (−9.48–−1.06)	6.016	0.014
4 weeks	73.33 ± 7.33AB	79.20 ± 8.01ABa	−5.87 (−9.68–−2.05)	9.069	0.003
12 weeks	74.90 ± 5.08AB	85.13 ± 4.80ABCa	−10.23 (−12.69–−7.77)	66.458	<0.001
24 weeks	74.97 ± 5.37AB	85.17 ± 5.88ABCa	−10.20 (−13.00–−7.40)	50.969	<0.001
χ^2^	97.331	3,020.319			
* P*	<0.001	<0.001			
Overall test					
Group point-in-time	χ^2^ = 15.46, *P* < 0.001, χ^2^ = 485.09, *P* < 0.001				
Group* point-in-time	χ^2^ = 48.43, *P* < 0.001				

(A) Compared with pre-treatment, *P* < 0.05; (B) Compared with 1 week after treatment, *P* < 0.05; (C) Compared with 4 weeks after treatment, *P* < 0.05; (D) Compared with 12 weeks after treatment, *P* < 0.05; (a) Compared with the control group, *P* < 0.05; All pairwise comparisons were adjusted using the Bonferroni correction.

GEE, generalized estimating equations model; χ^2^, Wald Chi-square.

### 3.5 Comparison of range of motion between two groups before and after treatment

GEE results revealed an interaction effect when comparing the range of motion (ROM) of knee joint flexion. Subsequent pairwise comparisons of simple effects showed no statistically significant difference in ROM between the two groups prior to treatment: the mean difference was 0.90 [95% confidence interval (CI): (−0.89–2.69), *P* = 0.325]. However, statistically significant between-group differences in ROM were noted at 1 week, 4 weeks, 12 weeks, and 24 weeks after treatment. Specifically, the mean differences (95% CIs) at these time points were −5.73 (−6.53 to −4.94), −6.53 (−7.39 to −5.67), −5.63 (−6.43 to −4.84), and −4.93 (−5.87 to −4.00), respectively, with all *P*-values < 0.05 (Table 5).

**Table 5:** GEE analysis of range of motion in the two groups before and after treatment.

Point-in-time	Group A (*N* = 60)	Group B (*N* = 60)	Differentials (95% CI)	χ^2^	*P*
Before treatment	120.73 ± 3.44	119.83 ± 3.76	0.90 (−0.89–2.69)	0.967	0.325
1 week	122.13 ± 1.31	127.87 ± 1.85Aa	−5.73 (−6.53–−4.94)	198.639	<0.001
4 weeks	122.83 ± 1.46B	129.37 ± 1.96ABa	−6.53 (−7.39–−5.67)	221.887	<0.001
12 weeks	123.93 ± 1.34ABC	129.57 ± 1.81ABa	−5.63 (−6.43–−4.84)	193.985	<0.001
24 weeks	125.20 ± 1.75ABCD	130.13 ± 2.00ABCDa	−4.93 (−5.87–−4.00)	107.232	<0.001
χ^2^	667.623	402.757			
* P*	<0.001	<0.001			
Overall test					
Group point-in-time	χ^2^ = 133.40, *P* < 0.001				
	χ^2^ = 516.03, *P* < 0.001				
Group* point-in-time	χ^2^ = 85.016, *P* < 0.001				

(A) Compared with pre-treatment, *P* < 0.05; (B) Compared with 1 week after treatment, *P* < 0.05; (C) Compared with 4 weeks after treatment, *P* < 0.05; (D) Compared with 12 weeks after treatment, *P* < 0.05; (a) Compared with the control group, *P* < 0.05; All pairwise comparisons were adjusted using the Bonferroni correction.

GEE, generalized estimating equations model; χ^2^, Wald Chi-square.

### 3.6 Comparison of clinical efficacy between two groups before and after treatment

Clinical efficacy was categorized into excellent response, good response, and non-response. Specifically, excellent response was defined as the complete disappearance or significant reduction of knee joint symptoms (e.g., pain, swelling, and stiffness) after treatment, with essentially normal joint mobility and the ability to perform daily life and work activities. Good response referred to a tendency toward alleviation of knee joint pain, stiffness, and swelling, with minimal impact on daily life and work. Non-response indicated no improvement in knee joint symptoms, no change in joint mobility, and persistent severe impairment of daily life and work. The total effective rate was defined as the sum of excellent and good responses.

Regarding clinical efficacy, there was a statistically significant difference in the total effective rate between the two groups (*P* < 0.05), indicating that compared with single hyaluronic acid sodium injection, intra-articular “triple injection” therapy for the knee joint exhibited significantly superior clinical efficacy, which was beneficial for relieving knee osteoarthritis (KOA)-related pain and restoring KOA-related function. When comparing the efficacy of the two groups within the same Kellgren-Lawrence (K-L) classification, the results showed no statistically significant difference in clinical efficacy for K-L grade II (*P* > 0.05). In contrast, a statistically significant difference was observed in clinical efficacy for K-L grade III (*P* < 0.05) (Tables 6–8).

**Table 6:** Comparison of the clinical outcomes of the two groups after treatment.

Clinical outcomes	Group A (*N* = 60)	Group B (*N* = 60)	Z	*P*
Ineffective	11 (36.7)	3 (10.0)		
Good effective	13 (43.3)	8 (26.7)		
Excellent effective	6 (20.0)	19 (63.3)		
Total effective rate (*n*, %)	19 (63.3)	27 (90.0)	3.475	<0.001

**Table 7:** Comparison of clinical outcomes between the two groups after treatment (K-L-II).

Clinical outcomes	Group A (*N* = 38)	Group B (*N* = 34)	Z	*P*
Ineffective	12 (31.6)	2 (5.9)		
Good effective	14 (36.8)	12 (35.3)		
Excellent effect	12 (31.6)	20 (58.8)		
Total effective rate (*n*, %)	26 (68.4)	32 (94.1)	2.001	0.066

**Table 8:** Comparison of clinical outcomes between the two groups after treatment (K-L-III).

Clinical outcomes	Group A (*N* = 22)	Group B (*N* = 26)	Z	*P*
Ineffective	10 (45.5)	4 (15.4)		
Good effective	12 (54.5)	4 (15.4)		
Excellent effect	0 (0.0)	18 (69.2)		
Total effective rate (*n*, %)	12 (54.5)	22 (84.6)	2.984	0.004

### 3.7 MOCART score between two groups before and after treatment

We evaluated the Magnetic Resonance Imaging (MRI)-based MOCART scores of all patients. The results showed no statistically significant difference in MOCART scores between Group A and Group B either before or after injection; additionally, intragroup comparisons of MOCART scores before and after injection in both groups also revealed no statistically significant differences. Therefore, this study demonstrated that the triple injection is safe in the short term for addressing potential cartilage damage (Tables 9, 10).

**Table 9:** Comparison of MOCART score in group A before and after treatment.

MOCART score	Before treatment	After treatment	*P1*	*P2*
Total points	48.97	49.26	0.52	0.18
Cartilage/bone defect volume filling	9.17	9.23	0.42	0.90
Integration with adjacent tissues	10.03	10.17	0.56	0.52
Repaired tissue surface integrity	3.67	3.70	0.57	0.67
Repaired tissue signal intensity	0	0.00	1.00	0.16
Bone defect or bone spicule presence	12.56	12.63	0.42	0.76
Bone marrow edema-like signal	5.23	5.16	0.33	0.37
Subchondral bone cyst formation	8.00	8.03	0.75	0.86

*P*1: Comparison of MOCART scores before and after treatment in **Group A** patients.

*P*2*:* Comparison of MOCART scores **before treatment** between Group A and B.

**Table 10:** Comparison of MOCART score in group B before and after treatment.

MOCART score	Before treatment	After treatment	*P*1	*P*3
Total points	49.63	50.10	0.18	0.15
Cartilage/bone defect volume filling	9.27	9.30	0.77	0.83
Integration with adjacent tissues	10.06	10.00	0.73	0.78
Repaired tissue surface integrity	3.73	3.76	0.71	0.85
Repaired tissue signal intensity	0.00	0.00	1.00	0.16
Bone defect or bone spicule presence	12.90	13.00	0.64	0.42
Bone marrow edema-like signal	5.53	5.60	0.69	0.23
Subchondral bone cyst formation	8.10	8.13	0.83	0.64

*P*1: Comparison of MOCART scores before and after treatment in **Group B** patients.

*P2:* Comparison of MOCART scores **after treatment** between Group A and B.

## 4 Discussion

KOA is a prevalent degenerative joint disease characterized by joint pain, disability, and loss of function (12). Currently, there is no curative treatment for KOA in clinical practice. For KOA patients who respond poorly to oral analgesics or anti-inflammatory drugs and suffer from moderate to severe pain, intra-articular drug injection into the knee joint is a viable therapeutic option. In recent years, the application of intra-articular drug injection in KOA treatment has gained increasing popularity (13). Compared with systemic administration and surgical treatment, intra-articular drug injection offers advantages such as high bioavailability, fewer systemic adverse reactions, and lower treatment costs (14).

The injection protocol used in this study was the “triple injection,” consisting of 0.3 mL compound betamethasone, 0.7 mL lidocaine, and 2 mL sodium hyaluronate. Sodium hyaluronate is composed of hyaluronic acid (HA), for which there are relatively few studies. It is widely distributed in both humans and animals, exerting multiple biological functions, including chondroprotection, anti-inflammatory effects, and joint lubrication, as well as protective effects on the meniscus (15). Intra-articular injection of sodium hyaluronate for KOA treatment improves joint lubrication, and reduces friction between articular cartilages, thereby alleviating joint pain, improving joint mobility. This therapy demonstrates significant efficacy in patients with early and mid-stage KOA (16–18). A single 5-week course of intra-articular sodium hyaluronate injection can relieve pain and improve joint function in KOA patients for up to 6 months, without serious side effects or complications (19). However, in clinical practice, it is commonly observed that the therapeutic effect of single-agent sodium hyaluronate injection is not significant in patients with more severe KOA, and combination injection with other drugs is usually required for intervention (19). However, in clinical practice, it is commonly observed that the therapeutic effect of single-agent sodium hyaluronate injection is not significant in patients with more severe KOA, and combination injection with other drugs is usually required for intervention.

Notably, primary KOA is typically associated with aseptic inflammation, which is the primary factor contributing to pain symptoms and disease progression (20). Studies have shown that intra-articular administration of lidocaine can reduce pain transmission in damaged peripheral nerves, thereby inhibiting pain sensitization, protecting already sensitized peripheral nerves in the injured area, and stabilizing neuronal cell membranes (21, 22). Literature reports indicate that a single intra-articular injection of 5 mL of 2% lidocaine into the knee joint cavity does not compromise chondrocyte viability, whether in healthy cartilage or KOA-affected cartilage (23). Intra-articular injection of local anesthetics is therefore widely used in the treatment of patients with painful knee KOA (24).

Regarding steroids, the “Management Guidelines for Hand, Hip and Knee Osteoarthritis (2019)” published by the American College of Rheumatology/Arthritis Foundation (25), recommends intra-articular steroid injections for KOA patients. Within the joint cavity, steroids exert a potent anti-inflammatory effect, aiding in alleviating KOA-related pain and promoting the recovery of knee function. They are indicated for the treatment of acute joint effusion and non-specific inflammation of surrounding soft tissues in KOA (26). The mechanism of action of glucocorticoids primarily involves inhibiting the production and release of inflammatory mediators, suppressing immune cell activity, and reducing the immune response (27). Studies have reported that the duration of pain relief following intra-articular glucocorticoid injection in the knee joint is at least 1 week (28), and can last up to 6 weeks (29), which is consistent with the findings of the present study. In a double-blind, randomized controlled trial, intra-articular injection of triamcinolone acetonide (10 mg) was found to be non-inferior to 40 mg triamcinolone acetonide in improving pain in KOA patients. Both doses significantly relieved pain and improved quality of life in symptomatic KOA patients. Compound betamethasone is a long-acting glucocorticoid with strong anti-inflammatory activity; additionally, intra-articular injection of 0.5–2 mL compound betamethasone can alleviate OA-related pain and stiffness within 2–4 h (30), which was the primary rationale for selecting betamethasone in this study. Andzie-Mensah et al. (31) conducted a single-blind randomized controlled study to compare the efficacy of betamethasone dipropionate and methylprednisolone acetate in the treatment of primary knee osteoarthritis [Kellgren-Lawrence (K-L) stages 2–4]. The results showed that both steroid injections alleviated symptoms; however, betamethasone dipropionate exhibited superior long-term efficacy compared with methylprednisolone acetate, providing sustained pain relief for more than 8 weeks. Wattanasirisombat et al. (32) performed a single-center, double-blind, randomized controlled trial to compare the efficacy of a single injection of long-acting corticosteroid (betamethasone) and medium-acting corticosteroid (triamcinolone) in KOA treatment. The results demonstrated that both the betamethasone and triamcinolone groups exhibited a significant reduction in resting Visual Analog Scale (VAS) pain scores starting from day 1, which persisted for up to 6 months. However, no significant differences were observed between betamethasone and triamcinolone in terms of resting VAS pain scores, functional scores, or performance-based outcomes at 6 months, highlighting the need for longer-term follow-up in the present study.

However, studies have shown that repeated use of corticosteroids can have adverse effects on articular cartilage (33). Corticosteroid use can also induce various other physiological reactions in the body, including disrupting water, salt, sugar, protein, and fat metabolism, impairing the body's resistance, hindering tissue repair, and delaying tissue healing (34). Additionally, some studies have reported that multiple short-term corticosteroid injections may cause cartilage damage and increase the risk of exacerbating KOA progression (35). Wernecke et al. (36) conducted a study and found that intra-articular corticosteroid injections at low doses and low frequencies (≤2–3 mg per dose or total cumulative dose of 8–12 mg in the body) exerted beneficial effects, whereas higher doses and frequencies (>3 mg per dose or total cumulative dose of 18–24 mg in the body) caused chondrotoxic effects and cartilage damage. Whitaker et al. (37) evaluated the safety and tolerability of co-injecting 2.5% polyacrylamide hydrogel with betamethasone disodium phosphate and betamethasone acetate into the healthy metacarpophalangeal joints of horses. The results showed that injecting 1 mL of betamethasone (6 mg/mL) into horses was safe for articular cartilage. Davis et al. (38) investigated chondrocytes treated with a combination solution of betamethasone sodium phosphate and betamethasone acetate (without benzalkonium chloride) at concentrations of 0.2, 0.6, 1, 3, and 6 mg/mL for 30 min. They found that no significant chondrocyte death was observed in the above concentration groups within 7 days after treatment; intra-articular injection of a low dose of betamethasone (<2.1 mg per dose) did not cause obvious cartilage damage or cell death, whereas injection of a higher dose (>2.1 mg per dose) led to cartilage protein loss and subsequent cartilage damage. Therefore, to minimize potential articular cartilage damage caused by betamethasone, the “triple injection” used in the observation group of this study contained a single low dose of compound betamethasone (0.3 mL, containing 1.5 mg of betamethasone dipropionate and 0.6 mg of betamethasone sodium phosphate).

Consistent with Davis et al. (38), this study also did not find that the triple injection caused cartilage damage. In their systematic review, Pirri et al. (39) clearly pointed out the potential cartilage damage that glucocorticoids and lidocaine may induce. Due to the lack of standardization in injection protocols and dosages, definitive conclusions cannot be drawn, and clinicians must remain vigilant about their potential hazards at all times. Furthermore, in a clinical randomized controlled study (40), the observation group and the control group received intra-articular injections of triamcinolone acetonide and normal saline once every 3 months, respectively. After 2 years of continuous injections, magnetic resonance imaging (MRI) revealed a 0.11 mm difference in knee articular cartilage thickness between the two groups, indicating that corticosteroids cause an annual cartilage loss of approximately 0.055 mm. Given that the thickness of knee articular cartilage is 3–5 mm, continuous intra-articular injections of corticosteroids once every 3 months would require 10 years to reduce articular cartilage thickness by 12.5%, a change that has a relatively minor impact on KOA progression. Regarding the safety of glucocorticoids, no statistically significant differences in K-L scores or total knee replacement rates were observed between patients who received glucocorticoid injections and those who did not after 5 years of follow-up (41, 42). In the study by Baker et al. (43), the chondrotoxicity of the combined use of local anesthetics, glucocorticoids, and hyaluronic acid was investigated. The results showed that this combination could produce a synergistic anti-inflammatory effect and counteract, to some extent, the side effects associated with single-drug use. Consistent with this, the present study also found that the triple injection achieved significantly superior efficacy compared with sodium hyaluronate alone. However, it should be clearly noted that betamethasone alone exerts anti-inflammatory and analgesic effects, while lidocaine has anesthetic effects. Whether the triple injection combination is more effective than betamethasone or lidocaine alone requires further research.

## 5 Limitations

This study has several limitations. First, the sample size included was relatively small, and this was a single-center study, which may introduce bias into the research results. Second, in this study, the observation group was treated with the “triple injection” (0.3 mL compound betamethasone + 0.7 mL lidocaine + 2 mL hyaluronic acid) for KOA, while the control group only received 2 mL of hyaluronic acid injection. The inconsistent injection doses between the two groups may potentially interfere with the evaluation of therapeutic efficacy. Third, WOMAC score, VAS score and HSS score used in this study are all subjective indicators. There are certain deviations in the assessment process of these scoring scales, and no objective indicators were included for comprehensive evaluation—for example, monitoring changes in inflammatory factors in knee synovial fluid before and after treatment, or conducting imaging assessments post-treatment. Fourth, this study adopted a single-blind design, which may lead to subjective bias in data collection by researchers. Fifth, the follow-up period of this study was relatively short, which may affect the stability of the results. Last but not least, it is worth noting that lidocaine and betamethasone were used as part of the intervention to mimic the typical clinical experience of patients; however, their use may affect the judgment of the triple injection's effectiveness.

## 6 Conclusion

Intra-articular injection of sodium hyaluronate and the “triple injection” are both effective therapeutic modalities for the early and mid-stage of KOA. Compared with sodium hyaluronate, the “triple injection” can more effectively relieve pain and improve knee joint function.

## Data Availability

The original contributions presented in the study are included in the article, further inquiries can be directed to the corresponding author.
